# Clinical characteristics of disseminated cryptococcosis in previously healthy children in China

**DOI:** 10.1186/s12879-017-2450-5

**Published:** 2017-05-22

**Authors:** Li-Wei Gao, An-Xia Jiao, Xi-Rong Wu, Shun-Ying Zhao, Yun Ma, Gang Liu, Ju Yin, Bao-Ping Xu, Kun-Ling Shen

**Affiliations:** 1 Respiratory Department, Beijing Children’s Hospital, Capital Medical University, Beijing, China; 2 China National Clinical Research Center for Respiratory Diseases, Beijing, China; 3 Department of Infectious Diseases, Beijing Children’s Hospital, Capital Medical University, Beijing, China

**Keywords:** Disseminated cryptococcosis, Healthy, Immunocompetent, Children

## Abstract

**Background:**

Disseminated cryptococcosis is a rare and fatal disease, and limited data exist regarding it in children. This study aimed to investigate the clinical characteristics of disseminated cryptococcosis in previously healthy children in China.

**Methods:**

Hospitalized patients with disseminated cryptococcosis were enrolled during January 1996 to December 2015 in Beijing Children’s Hospital, Capital Medical University, China. Data on clinical manifestations, laboratory tests, treatment, and prognosis were evaluated.

**Results:**

A total of 52 pediatric patients with no underlying disease were enrolled, including 38 boys and 14 girls. Only 10 cases had a history of exposure to pigeon droppings. Fever, cough, and hepatomegaly were 3 main manifestations of disseminated cryptococcosis. However, headache was more common in patients with central nervous system (CNS) invasion than in patients with non-CNS invasion (*P* < 0.05). Lung (96.2%, 50/52) was the most commonly invaded organ, but only 9.6% (5/52) of patients had respiratory signs. The most common findings on chest imaging were hilar or mediastinal lymphadenopathy (46.8%, 22/47), and nodules (44.7%, 21/47), including small nodules in a scattered distribution (57.1%, 12/21) or miliary distribution (42.9%, 9/25), especially localized in subpleural area. Subsequent invasion occurred in the CNS, abdomen lymph nodes, liver, spleen, peripheral lymph nodes, and skin. In all patients, 42.3% (22/52) and 51.9% (27/52) had elevated eosinophils or IgE, respectively. The positive rate of serum cryptococcal antigen was higher, especially in patients with CNS invasion (approximately 83.3%), than with other primary methods used for pathogen detection, including cerebrospinal fluid (CSF) cryptococcal antigen, cultures of blood, bone marrow, or CSF, and CSF ink staining. The overall mortality rate of pediatric patients in our study was 11.5% (6/52). Some cases had long-term sequela, including hydrocephalus, cirrhosis, or blindness.

**Conclusions:**

Disseminated cryptococcosis can occur in previously healthy or immunocompetent children in China. Lung and CNS were most commonly invaded by this disease. Furthermore, most cases usually showed no obvious or specific symptoms or signs, and therefore pediatricians should pay more careful attention to identify this disease.

## Background

Cryptococcus belongs to capsulated yeast and can cause an invasive and fatal disease. Most occurs in adults aged 20 to 50 years old, approximately 1%–5% of the population, while in children the incidence is less than 1% [1]. When cryptococcus is inhaled into the respiratory tract, it might localize in immunocompetent hosts. Once individuals show immune defects, cryptococcus could disseminate hematogenously to any part of the body, including the central nervous system (CNS), lymph nodes, liver, spleen, kidney, and skin, especially in patients with profound cellular immune deficiency [2]. If 2 or more are invaded, patients would be diagnosed with disseminated cryptococcosis [3]. Joshi et al. [4] reported that cryptococcosis usually occurs in immunocompromised subjects, accounting for nearly 80% of patients with cryptococcal infection, especially in those with human immunodeficiency virus (HIV) infection. This is followed by primary immunodeficiencies, diabetes, and requiring glucocorticosteroid therapy or organ transplantation. On the contrary, studies [5, 6] in China demonstrated that 50–77% of patients with cryptococcal meningitis exhibited normal immune function. However, disseminated cryptococcosis is rare in immunocompetent children, and is mostly localized in the brain or lungs [7]. It is usually misdiagnosed as tuberculosis or other diseases. Some studies were limited to case reports or only several cases [8]. Furthermore, the spectrum of clinical manifestations and laboratory tests were not reported systematically. Therefore, we conducted this study to analyze the clinical characteristics of disseminated cryptococcosis in previously healthy children in China.

## Methods

### Study subjects

We retrospectively reviewed hospitalized patients less than 18 years old with disseminated cryptococcosis and treated between January 1996 and December 2015 in Beijing Children’s Hospital, Capital Medical University, China. Collected information included the following: (1) demographic features of the patients, history of exposure to pigeon droppings, course of disease and past medical history; (2) clinical features and symptoms; (3) laboratory tests, including full blood cell tests, liver and renal function tests, CD4+ and CD8+ T cells subsets, pathogen tests, imaging, ultrasounds, and pathology; and (4) treatment and prognosis. Information on the prognosis of pediatric patients was collected using telephone follow-up.

### Definitions

#### Diagnostic criteria of disseminated cryptococcosis

The diagnostic criteria of disseminated cryptococcosis have been defined as 2 or more non-adjacent organs being simultaneously affected with cryptococcosis [3]. Cryptococcal infection was validated by positive culture of cryptococcus from blood or cerebrospinal fluid (CSF), cryptococcal antigens of blood and/or CSF, or CSF ink staining. Pathological diagnosis of cryptococcosis is based on positive staining methods of pathological specimens for cryptococcus, including hematoxylin and eosin, periodic acid Schiff, and methenamine silver staining.

#### Cryptococcal antigen assay and drug sensitive test

Cryptococcal antigen assay was conducted using the Immy Latex-Crypto Antigens (Immuno-Mycologics, Inc.). Blood samples were cultured with chromogenic agar medium (CHRO) and identified by an API-20CAUX strip for yeast-like fungi. Then, we conducted a drug sensitivity test by ATB-FUNGU Strip. These drugs included amphotericin B, fluconazole, itraconazole, and 5-flucytosine.

#### Previously healthy children

In this study, we defined previously healthy children as patients with no underlying disease, including acquired immune deficiency syndrome (AIDS), prolonged corticosteroid usage, immunodeficiencies, organ transplantation, advanced malignancy, and diabetes. Furthermore, all pediatric patients were with normal numbers of CD4+ T cells, CD8+ T cells, B cells, and NK cells, and had normal levels of IgG, IgA, and IgM.

#### Statistical analysis

All data were analyzed using SPSS version 19.0 software. Continuous data are presented as the mean ± standard deviation or range. Proportions were compared by the Chi-squared or Fisher’s exact test, and continuous variables within 2 groups were compared using the independent *t* test. All tests were 2-tailed, and *P* < 0.05 was considered statistically significant.

## Results

### Demographic features of patients

Fifty-two children with disseminated cryptococcosis were enrolled in this study, 38 of which were male and 14 female, for a male-to-female ratio of 2.7:1. The median age was 4.3 years, ranging from 1.3 years to 11 years. The age distribution of these patients is shown in Table 1. The course of the disease before hospitalization ranged from 18 days to 5 years, and the median was 40 days. Some patients had been misdiagnosed as having tuberculosis, bacterial and virus infections, or eosinophilia before hospitalized. Only 10 (19.2%) cases had a history of exposure to pigeon droppings.

**Table 1:** Clinical features of 52 pediatric patients with disseminated cryptococcosisa

Clinical features	Total %(n/N)	CNS invasion %(n/N)	Non-CNS invasion %(n/N)	*P*
Age (year)				0.600
< 3	21.2 (11/52)	22.2(8/36)	18.8(3/16)	
3–5	42.3 (22/52)	38.9(14/36)	50.0(8/16)	
≥ 5	36.5 (19/52)	38.9(14/36)	31.3(5/16)	
Male	73.1 (38/52)	75.0(27/36)	68.8(11/16)	0.639
History of exposure to pigeon droppings	19.2 (10/52)	22.2(8/28)	12.5(2/14)	0.411
Main symptoms and signs				
Fever	100 (52/52)	100 (36/36)	100 (16/16)	-
Cough	53.8 (28/52)	55.6(20/36)	50.0(8/16)	0.710
Hepatomegaly	51.9 (27/52)	47.2(17/36)	62.5(10/16)	0.308
Meningeal irritation	34.6 (18/52)	100 (36/36)	0	0.000
Headache	28.8 (15/52)	38.9(14/36)	6.3(1/16)	0.017
Lymphadenopathy	27.0 (14/52)	33.3(12/36)	12.5(2/16)	0.118
Splenomegaly	27.0 (14/52)	16.7(6/36)	50.0(8/16)	0.012
Vomiting	23.1 (12/52)	30.6(11/36)	6.3(1/16)	0.056
Abdominal pain	19.2 (10/52)	22.2(8/36)	12.5(2/16)	0.411
Rash	11.5 (6/52)	13.9(5/36)	6.3(1/16)	0.426
Lung auscultation	9.6 (5/52)	11.1(4/36)	6.3(1/16)	0.583
Seizure	9.6 (5/52)	13.9(5/36)	0	0.117
Jaundice	7.7 (4/52)	8.3(3/36)	6.3(1/16)	0.795
Invaded organs				
Lung	96.2 (50/52)	97.2(35/36)	93.8(15/16)	0.548
CNS	69.2 (36/52)	100 (36/36)	0	0.000
Abdomen lymph nodes	67.3 (35/52)	58.3(21/36)	87.5(14/16)	0.039
Liver	63.5 (33/52)	63.9(23/36)	62.5(10/16)	0.924
Spleen	50.0 (26/52)	47.2(17/36)	56.3(9/16)	0.548
Peripheral lymph nodes	38.5 (20/52)	38.9(14/36)	37.5(6/16)	0.924
Skin	11.5 (6/52)	13.9(5/36)	6.3(1/16)	0.426
Blood test				
Increased CRP	100 (52/52)	100 (36/36)	100 (16/16)	-
Increased ESR	100 (52/52)	100 (36/36)	100 (16/16)	-
Leukocytosis	92.3 (48/52)	91.7(33/36)	93.8(15/16)	0.795
IgE	51.9 (27/52)	58.3(21/36)	37.5(6/16)	0.135
Eosinophilia	42.3 (22/52)	36.1(13/36)	56.3(9/16)	0.175
Abnormal liver function	38.5 (20/52)	38.9(14/36)	37.5(6/16)	0.924
Pathogen test				
Serum cryptococcal antigen	73.1 (38/52)	83.3(30/36)	50.0(8/16)	0.012
Blood culture	46.2 (24/52)	58.3(21/36)	18.8(3/16)	0.008
CSF cryptococcal antigen	42.9 (21/49)	58.3 (21/36)	0	0.000
CSF ink staining	38.8 (19/49)	52.8 (19/36)	0	0.000
CSF culture	32.4 (11/49)	30.6 (11/36)	0	0.013
Pathology	100 (19/19)	19.4(7/36)	75.0(12/16)	0.000
Chest radiographic abnormalities	90.4 (47/52)	88.9(32/36)	93.8(15/16)	0.583
Abdominal examination abnormalities	73.1 (38/52)	61.1(22/36)	100 (16/16)	0.004
Neurology imaging abnormalities	40.4 (21/52)	58.3(21/36)	0	0.000

### Clinical features

All 52 patients (100%) had fever (defined as a temperature above 37.3 °C), and the mean peak temperature was 39.3 ± 0.6 °C (range: 38.5–40.5 °C). The incidences of cough, hepatomegaly, and headache were 53.8% (28/52), 51.9% (27/52), and 28.8% (15/52), respectively. Most coughs were mild and dry (75.0%, 21/28). Splenomegaly, lymphadenopathy, rash, and other symptoms were also seen in these patients. In addition, 2 patients presented with facial paralysis, 2 with loss of consciousness, and 1 with diarrhea and blindness. Hospitalization time ranged from 2 days to 109 days, with an average of 42.4 ± 27.2 days. Compared with the non-CNS invasion group, pediatric patients in the CNS invasion group had a higher rate of headache (*P* < 0.05), but lower rate of splenomegaly (*P* < 0.05) (see Table 1).

In these 52 patients, 15.4% (8/52) had invasion of 2 organs, while 84.6% (44/52) had invasion of 3 or more organs. Four cases developed invasion of 7 organs (the highest number of invaded organs). In this study, lung (96.2%, 50/52) was the most commonly invaded organ, of which 22 patients were without respiratory symptoms, but with fever or headache or other symptoms. The second most commonly invaded organ was the CNS (69.2%, 36/52), which manifested with headache, fever, nausea, vomiting, and irritability. Meanwhile, 33.3% (12/36) had no nervous system manifestations but other non-CNS related symptoms. In this study, cranial nerve was invaded in 5 patients (9.6%), including the facial nerve in 2 cases, the oculomotor nerve in 2 cases, and the abducens nerve in 1 case. Cryptococcus was also susceptible to invading the abdomen lymph nodes, liver, spleen, and peripheral lymph nodes, accounting for 67.3%, 63.5%, 50.0%, and 38.5% respectively (Table 1). However, not all patients with liver/spleen invasion had hepatosplenomegaly. Enlargement of abdominal lymph nodes could be palpated in only 4 cases, and the largest mass was about 8 × 7 × 3 cm. Then using abdominal ultrasound, the large masses were recognized as adhesive lymph nodes. Cutaneous manifestations of cryptococcosis including herpes and nodules were found in 6 cases (11.5%). Additionally, 9 patients had bone marrow invasion, 3 patients had kidney invasion, 2 patients had intestinal tract and pericardium invasion, and 1 case had chest wall invasion.

### Laboratory findings

Forty-eight patients had leukocytosis with an average of 22.1 ± 8.9 × 10^9^/L with predominant neutrophils, ranging from 10.5 × 10^9^/L to 45.75 × 10^9^/L, of which 61.0% (32/52) white blood counts (WBC) greater than 20 × 10^9^/L. Twenty-two cases (42.3%) had an elevated percentage of eosinophils, ranging from 8.1% to 57% (normal range: 0–5%). Meanwhile, decreased hemoglobin, ranging from 63 g/L to 83 g/L, was detected in 26.9% (14/52) of patients. C-reactive protein (CRP) and erythrocyte sedimentation rate (ESR) of all patients were increased, and ranged from 11 mg/L to 273 mg/L (average of 86.6 ± 70.9 mg/L), and 28 mm/h to 140 mm/h (average of 52.0 ± 36.3 mm/h), respectively. Sixteen (30.8%) patients’ CRP was higher than 100 mg/L, and 14 (26.9%) patients’ ESR was higher than 100 mm/h. Furthermore, IgE levels increased in 51.9% (27/52) of patients, and the highest was more than 2000 IU/L; however, this decreased quickly to normal levels after anti-fungal treatment. The additional laboratory tests had been performed in patients with high levels of eosinophils and IgE in order to exclude the other causes such as parasitic infection, eosinophilic lung diseases, and hyperimmunoglobulin E syndrome. Levels of IgG, IgA, and IgM were normal, as were the numbers of CD4+ T cells, CD8+ T cells, B cells, and NK cells. The percentage of CD4+ T cells and CD8+ T cells was slightly lower in just 4 cases. Furthermore, HIV was negative in all patients. Lastly, there was no evidence of immunodeficiencies in any patients.

The positive rates of serum cryptococcal antigen and blood culture were 73.1% (38/52) and 46.2% (24/52), respectively, and both were higher in patients with CNS invasion than in patients without CNS invasion (*P* < 0.05). Cryptococcal antigens in blood decreased slowly, a change that was detectable even 2 years later in 1 patient. In this study, 49 patients underwent lumbar puncture, and the positive of cryptococcal antigens, ink staining, and culture of cryptococcus in CSF samples were 42.9% (21/49), 38.8% (19/49), and 32.4% (11/49), respectively; these values were especially higher in patients with CNS invasion (*P* < 0.05) (Table 1). Furthermore, the positive rate of bone marrow culture was 28.1% (9/32), and sputum culture was positive in 3 cases. Finally, drug sensitivity tests showed that the fungus was sensitive to amphotericin B, fluconazole, itraconazole, and 5-flucytosine.

### Pathology

**Fig. 1:**
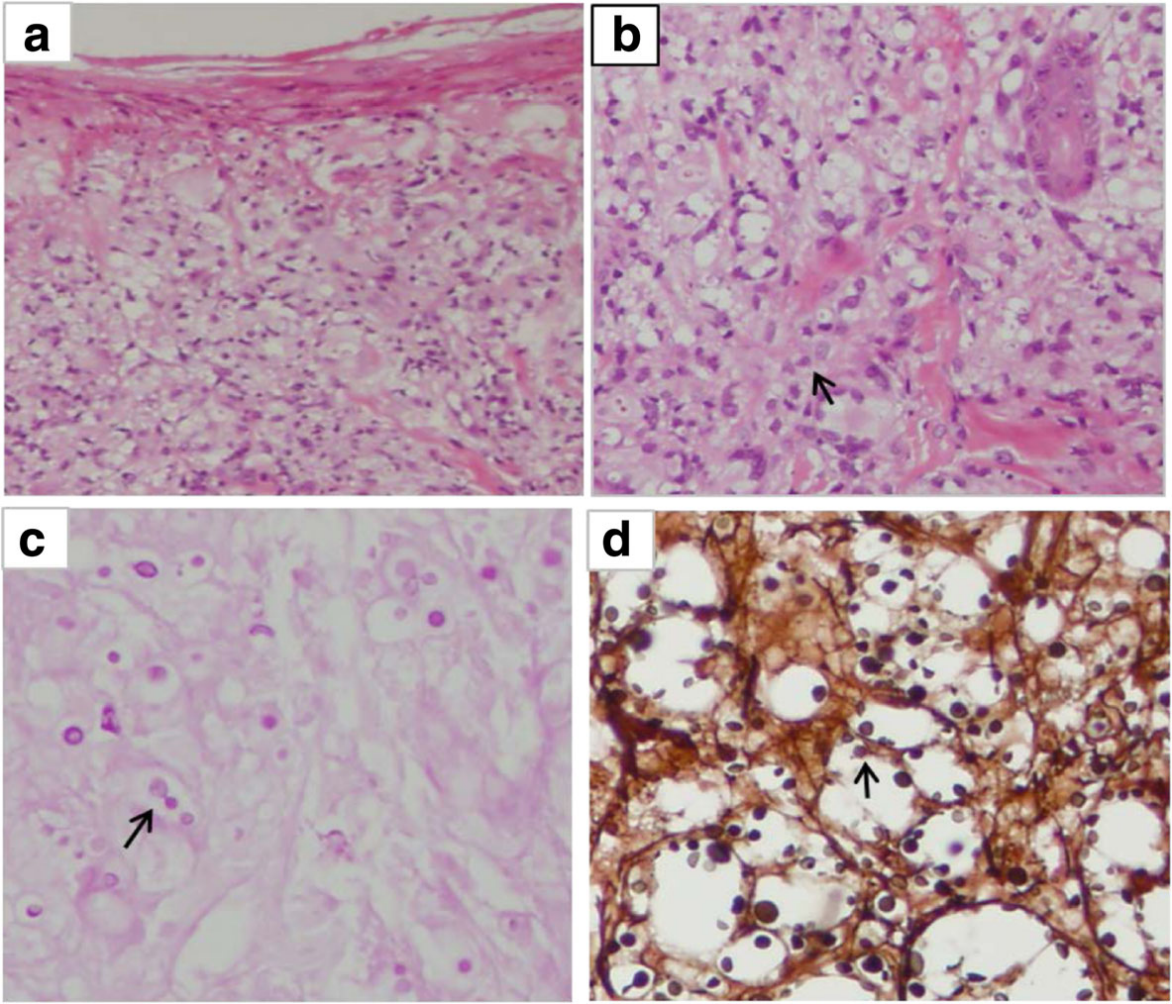
Skin biopsy was performed. **a**&**b** A specimen showed epithelioid cell granulomas with multinucleated giant cells and many yeast-like organisms (hematoxilin and eosin). **c** Positive Periodic acid-Schiff. **d** Positive Methenamine silver staining

Pathological examination was conducted in 19 patients, including that of the abdominal lymph node (*n* = 7), cervical lymph node (*n* = 6), liver (*n* = 2), and skin (*n* = 4). Results revealed structural damage, multifocal necrosis, abscesses, granulomatous inflammation, and cryptococcus infiltration. Periodic acid Schiff and methenamine silver staining were positive in all cases (Fig. 1). Two cases underwent surgical exploration and biopsy. An additional case showed hepatomegaly with yellowish brown nodules, lymphadenopathy around the hepatic hilar region, and splenomegaly with congestion; histopathologic examination showed cryptococcus infiltration. Two months later, this patient developed cirrhosis. Another case showed edematous mesentery with multiple rough and gray mesenteric lymphadenopathies, part of them adhering together, which was similar to tuberculosis. However, histopathologic examination showed massive hyperplasia of granuloma nodules and lots of cryptococcal spores in macrophages, with positive periodic acid Schiff and methenamine silver staining.

### Imaging and ultrasound

**Fig. 2:**
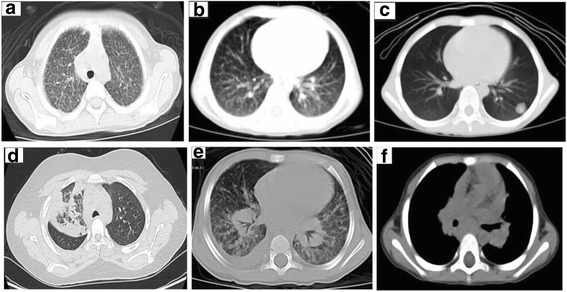
Chest computed tomography showed patterns of different type. **a** Bilateral miliary pattern. **b** Scatter small nodules distribution. **c** Isolated nodule localized in subpleura. **d** Patchy lesions in right lung. **e** Interstitial changes and hilar lymphadenopathy. **f** Mediastinal and hilar lymphadenopathy

Chest radiographic abnormalities were seen in 47 cases, and the most common findings were hilar or mediastinal lymphadenopathy (46.8%, 22/47) and nodules (44.7%, 21/47), including small nodules in a scatter distribution (57.1%, 12/21) or miliary distribution (42.9%, 9/21), and especially localized in subpleural area. Other findings displayed patchy shadows (38.3%, 18/47), mostly in the right upper lobe (*n* = 6), interstitial opacities (21.3%, 10/47), and pleural thickening (8.5%, 4/47) (Table 2 and Fig. 2). Some patients had more than 1 radiographic pattern. Additionally, axillary lymphadenopathy was seen in 3 cases, calcification of lymphadenopathy was detected in 1 case, and 1 case showed a mass with cavity.

**Table 2:** The abnormal imaging in 47 patients with disseminated cryptococcosis in chest radiographs

Radiographic pattern	%(n/N)
Hilar or mediastinal lymphadenopathy	46.8 (22/47)
Nodules	44.7 (21/47)
Scatter	57.1 (12/21)
Military	42.9 (9/21)
Patchy shadows	38.3 (18/47)
Interstitial opacities (*n* = 6)	21.3 (10/47)
Pleura thickening (*n* = 4)	8.5 (4/47)

**Fig. 3:**
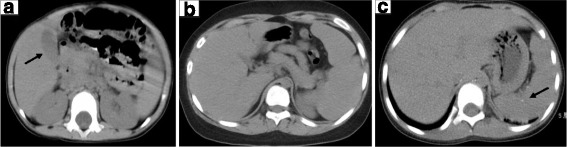
Abdominal CT scan. **a** Parenchymal low density lesions in the liver. **b** Lymphadenopathy in abdomen. **c** Calcification in spleen

All pediatric patients with disseminated cryptococcosis underwent abdominal ultrasound or computed tomography (CT) scan, and 44 patients had abnormal results. Hepatomegaly (61.4%, 27/44) and splenomegaly (31.8%, 14/44) were the most common findings. Among these patients, 15 had parenchymal low-density lesions both in the liver and spleen, while an additional 3 and 5 cases had diffuse nodules in the liver and spleen, respectively (Fig. 3). Lymphadenopathy in the abdomen was another common finding, including in the mesentery (50.0%, 22/44) (with calcification in 1 case, and liquefied necrosis in 2 cases), retroperitoneal space (22.7%, 10/44) (with calcification in 1 case), hepatic portal (15.9%, 7/44), abdominal para-aortic (9.1%, 4/44), splenic hilum region (4.5%, 2/44), and head of the pancreas (2.3%, 1/44). In addition, 3 cases presented with ascites, and 2 cases with gallbladder swelling or renal impairment, respectively (Table 3).

**Table 3:** The abnormal imaging in 44 patients with disseminated cryptococcosis in abdominal ultrasound or CT scan

Abnormal fingdings	%(n/N)
Hepatomegaly	61.4 (27/44)
Splenomegaly	31.8 (14/44)
Lymphadenopathy in abdomen	
Mesentery	50.0 (22/44)
Retroperitoneal	22.7 (10/44)
Hepatic portal	15.9 (7/44)
Ascites	6.8 (3/44)
Gallbladder swelling and	4.5 (2/44)
Renal impairment	4.5 (2/44)

**Fig. 4:**
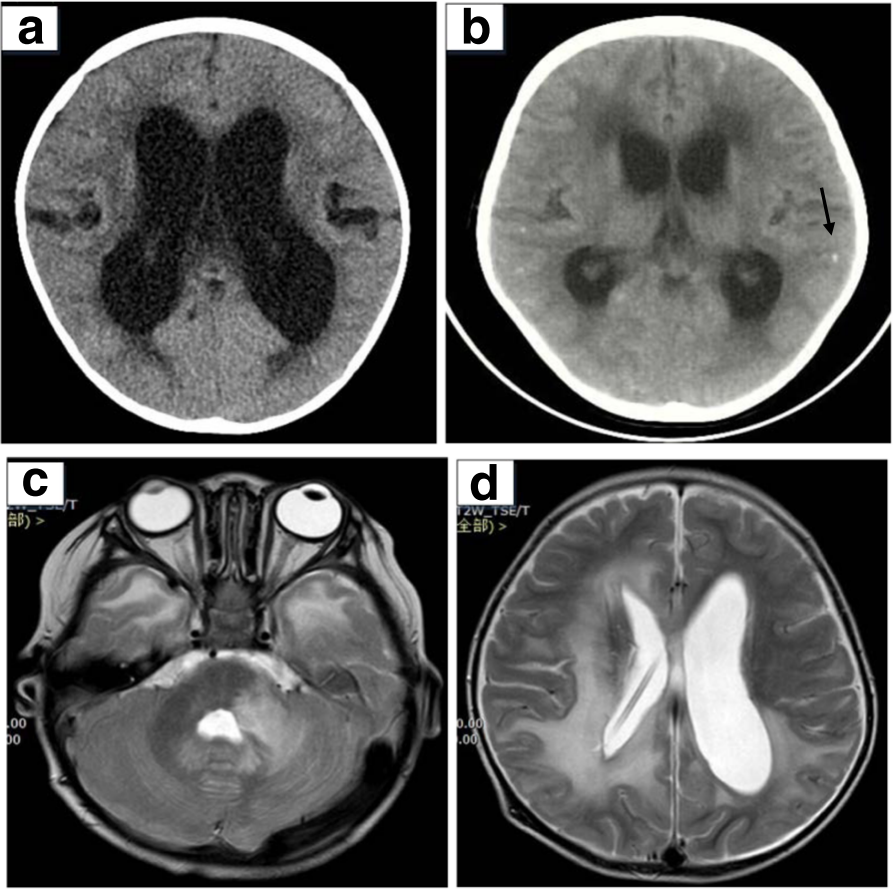
Cranial CT scan and MRI. **a** Hydrocephaly. **b** Calcification spots in brain parenchyma. **c** Abnormal signals in cerebral parenchyma. **d** Improved hydrocephaly after treated by lateral external ventricular drain

In patients with CNS invasion, 58.3% (21/36) of cases had abnormal neurology imaging. Among them, 52.4% (11/21) had hydrocephalus, including communicating hydrocephaly in 7 cases and non-communicating hydrocephaly in 4 cases. Furthermore, 38.1% (8/21) of cases had ventricular dilatation, 28.6% (6/21) had low-density lesions in the brain parenchyma, 23.8% (5/21) had meningeal enhancement, 14.3% (3/21) had calcification in the brain parenchyma, and 4.8% (1/21) had intracranial venous sinus thrombosis (Fig. 4). Additionally, vascular ultrasonic inspection showed 2 cases with vena iliaca communis thrombosis, and 1 case with iliofemoral vein thrombosis.

### Treatment and outcome

Forty-eight cases received anti-fungal treatment. Twenty-two patients used amphotericin B (0.7–1.0 mg/kg/d), 5-flucytosine (100 mg/kg/d), and fluconazole (6 mg/kg/d) in the initial treatment. For other treatment strategies, see Table 4. Then, oral fluconazole (6 mg/kg/d, for about 6 months to 12 months) was used for sustaining treatment in 36 patients. In addition, 4 patients with CNS cryptococcosis underwent intrathecal administration of amphotericin B, while 3 patients received ventricular drainage. After treatment, the condition of most patients improved, and the levels of WBC, CRP, ESR, eosinophils, and IgE decreased. The treatment duration of patients with CNS invasion (up to 29 months) was longer than in patients without CNS invasion (ranging from 1 to 7 months). Most patients treated with amphotericin B had good tolerance, except for 3 patients who had high fever and chills, and 2 who had elevated aminotransferase and neutropenia, respectively. The overall mortality rate of pediatric patients in our study was 11.5% (6/52), which was lower than in patients with CNS invasion (16.7%, 6/36).

**Table 4:** The initial treatment and response to therapy in 48 patients with disseminated cryptococcosis

Drug for initial treatment	Total cases (n)	Response to therapy	
		Improved	Died
fluconazole + amphotericin B+ 5-flucytosine	22	19	3
fluconazole	9	9	0
fluconazole + 5-flucytosine	6	6	0
fluconazole + amphotericin B	5	5	0
amphotericin B + 5-flucytosine	5	5	0
amphotericin B	1	1	0

## Discussion

Cryptococcosis is a rare and fatal disease that preferentially infects immunosuppressed hosts. A previous study [1] reported that the rate of cryptococcal infection in immunosuppressed patients (5–10%, up to 30% in patients with AIDS) was higher than in immunocompetent patients (less than 5% in adults, and less than 1% in children). However, Zhu et al. [9] reported that more than 60% of pediatric patients with cryptococcosis in China had normal immune functions. Luo et al. [10] also reported that nearly 70% of children with cryptococcosis were immunocompetent subjects and had no underlying illness or risk factors. Furthermore, Yuchong et al. [11] analyzed 8796 patients with cryptococcosis in mainland China from 1985 to 2010 and found that only 15.7% were HIV positive. This finding is interesting because the patients in our study were all HIV negative with no obvious immune deficiency. Additionally, Thompson and Chan [12–14] demonstrated that serotype A and D are more commonly detected in immunocompromised individuals or patients with a history of exposure to pigeon droppings, while serotype B and C (common seen in China) are usually observed in patients without significant immunosuppression. However, whether these previously healthy children in this study with normal levels of IgA, IgG, and IgM, and normal numbers of CD4 + T cells, CD8 + T cells, B cells, and NK cells in our study were real immunocompetent individuals is unknown and need further investigation.

In our study, the median age of children with disseminated cryptococcosis was 4.7 years old, and 63.5% of patients were less than 5 years old. This was similar to Luo et al.’s [10] study, but different from the study conducted by Joshi et al. [4] (where the median age was 12 years old). In addition, Goldman et al. [15] showed that most cases with cryptococcosis had pigeon dropping exposure; conversely, in our study, only 19.2% of patients had pigeon exposure. Therefore, if pediatric patients do not have a history of pigeon dropping exposure, the pediatrician should not omit a diagnosis of disseminated cryptococcosis.

In our study, fever, cough, and hepatomegaly were the 3 most common manifestations, while in the study by Severo et al. [16], headache, fever, vomiting, and neck pain were more common because most cases were cryptococcal meningitis. The lung was the most commonly invaded organ in our study, but respiratory symptoms were not obvious. This was inconsistent with chest X-ray or CT scan. Similar results were demonstrated by Suwatanapongched et al. [17]. Diffused miliary or scattered small nodules mainly in subpleural areas were the most common imaging features in our study, which is consistent with the results by Qu et al. [18]. In addition, studies by Xie et al. [19, 20] showed that cavitations within nodules/masses were more commonly seen in immunocompromised patients, especially in AIDS patients, while air bronchograms were more commonly seen in immunocompetent patients. Hilar or mediastinal lymphadenopathy was another common imaging finding in our study, which is relatively rare in immunocompetent patients and was reported in a previous case study [21]. Since diffused miliary and hilar or mediastinal lymphadenopathy are also 2 typical features of tuberculosis, pediatricians should pay attention to distinguishing between chronic pulmonary cryptococcosis and tuberculosis. The second most commonly invaded organ was the CNS (69.2%, 36/52) in our study. In contrast, Kaur et al. [22] showed that the CNS was the most commonly invaded site in pediatric AIDS with cryptococcosis, and it was related to CNS tissue tropism of cryptococcus. Therefore, when considering disseminated cryptococcosis, lumbar puncher should be done even if there are no significant signs of CNS invasion. Additionally, Shih et al. [23] reported that because of robust immune responses, immunocompetent patients exhibited more severe neurological complications than immunocompromised patients, such as hydrocephalus and seizure. They also showed that immunocompetent patients usually presented with longer symptom durations, typical meningeal signs, and neuroimaging findings. Meanwhile, immunocompromised patients usually manifested with high fever and parenchymal lesions in the brain. In our study, common neuroimaging findings were hydrocephalus, multiple intraparenchymal lesions, and ventricular dilatation; these findings are similar to those by Tan et al. [24]. In addition, Zhu et al. [25] showed that more than 70% of patients with cryptococcal meningitis were misdiagnosed, most as tuberculosis-associated diseases. However, different from tubercular meningitis, obliteration of basilar cistern and meningeal enhancement were less common in cryptococcal meningitis. Furthermore, cranial nerve impairment and intracranial venous sinus thrombosis were detected in our study, which was rarely reported before [6].

Besides lung and brain invasion, Dromer [26] demonstrated that the incidence of abdominal, renal, lymph node, and skin invasion (1.2%, 1.9%, 1.7%, and 0.4%, respectively) were lower than the results of the present study. However, in our study, nearly 67.3% of patients had abdominal lymph nodes invasion, including of the mesentery, hepatic portal, and abdominal para-aortic lymph nodes, and some of them were accompanied with calcification, which is rarely reported [27]. Several cases presented with abdominal pain or discomfort were misdiagnosed as tuberculosis because abdominal cryptococcosis has similar symptoms or pathologic results as tuberculosis. The incidence of the invasion of the liver, spleen, peripheral lymph nodes, and skin were relatively rare in our study. Poojary et al. [28] showed that cutaneous cryptococcosis may be present for 2 to 8 months before development of systemic signs of infection, and therefore this time period could provide a window of opportunity for treatment before fatal effects of dissemination occur. Furthermore, invasion of bone marrow, the intestinal tract, kidney, and pericardium by cryptococcal infection can be found in our study.

In this study, increased WBC (especially more than 20 × 10^9^/L), and high levels of CRP and ESR were common in patients with disseminated cryptococcosis. Furthermore, 42.3% and 51.9% of patients had elevated eosinophil and IgE, which is similar to results by Bassetti et al. [29, 30]. However, after treatment with anti-fungal drugs, levels of eosinophil and IgE decreased quickly; this was in line with results by Zhu et al. [9]. This could be explained by Th2 cell-mediated immune response [31]. Cryptococcal antigen tests, ink smears, culture, or pathological methodologies to confirm cryptococcus existence in specimens of blood, CSF, other tissues or body fluids were important diagnostic evidence. Antinori et al. [32] demonstrated that the sensitivity and specificity of cryptococcal capsular polysaccharide antigens in the diagnosis of cryptococcosis can reach to 96%. In our study, the positive rate of serum cryptococcal capsular polysaccharide antigens was 73.1%, which was even higher in patients with CNS invasion (reaching 83.3%). However, the positive rate of other methods was lower. Therefore, once disseminated cryptococcosis is suspected, these methods should be done repeatedly and combined. However, Lee et al. [33] showed that immunocompromised patients had higher serum cryptococcal antigen titers, and higher positive rates of fungal cultures from blood and CSF.

In our study, the clinical isolates in Chinese pediatric patients were sensitive to amphotericin B, fluconazole, itraconazole and 5-flucytosine. Chan et al. [13, 34] found that *Cryptococcus neoformans* predominantly infected immunocompetent individuals. The 2010 IDSA Clinical Practice Guidelines for the management of cryptococcal meningitis in non-HIV infected and non-transplant patients recommend induction therapy with amphotericin B (0.7–1.0 mg/kg/d) plus 5-flucytosine (100 mg/kg/d) for 2 weeks, followed by fluconazole (6 mg/kg) for a minimum of 8 weeks, and then maintenance therapy with fluconazole for at least 6 to 12 months [35]. Zhu et al. [9] pointed out that intrathecal administration of amphotericin B was an effective adjunctive treatment for many cryptococcosis patients in China. However, there are no well-controlled studies to clarify the role of intrathecal amphotericin B in the management of cryptococcal meningitis. Furthermore, the usage of intrathecal amphotericin B is not recommended in the 2010 IDSA guidelines. Patients with CNS invasion usually have a longer-term therapy than those without CNS invasion, which is consistent with these recommendations [35]. However, managing cryptococcosis remains a challenging issue, and how to determine the course of treatment remains unknown. Additionally, Zhu et al.’s [9] study demonstrated that immunocompetent individuals with cryptococcosis exhibited similar treatment responses and prognosis as immunocompromised patients; however, this should be confirmed by studies using a larger sample size. Most patients were cured in the present study. The mortality rate (11.5%) in our study was lower than that reported in a previous study (39.13%) [10]. This might be attributed to the lung being the most commonly invaded organ in our study. During follow-up, we found that some of the patients had severe sequela, including hydrocephalus, cirrhosis, and blindness. Liao et al. [36] reported that initial consciousness level, hydrocephalus, high CSF antigen titers, underlying diseases, non-amphotericin, B-based initial therapy, and delayed diagnosis (>120 days) were risk factors of poor prognosis. Therefore, pediatricians should pay attention to these indexes.

## Conclusions

Disseminated cryptococcosis can be seen in previously healthy or immunocompetent children in China. The lung and CNS were the 2 most commonly invaded organs in this study. Most cases showed no specific clinical manifestations, or were misdiagnosed as tuberculosis or other diseases. The prognosis of children with disseminated cryptococcosis is poor, but if diagnosed early, the majority cases could be managed successfully.

## Abbreviations

AIDS: Acquired immune deficiency syndrome
AmB: amphotericin B deoxycholate
CNS: Central nervous system
CRP: C-reactive protein
CSF: Cerebrospinal fluid
ESR: Erythrocyte sedimentation rate
MRI: Magnetic resonance imaging
WBC: White blood cell
